# Gender Difference in the Impact of Total Energy Intake on the Association between Low Fiber Intake and Mental Health in Middle-Aged and Older Adults

**DOI:** 10.3390/nu16162583

**Published:** 2024-08-06

**Authors:** Sinyoung Cho, Minseon Park

**Affiliations:** 1 Department of Family Medicine, Seoul National University Hospital, 101 Daehakro, Yeongun-dong, Jongno-gu, Seoul 03080, Republic of Korea; sinyoungcho@snu.ac.kr; 2 Department of Preventive Medicine, Seoul National University College of Medicine, 103 Daehakro, Yeongun-dong, Jongno-gu, Seoul 03080, Republic of Korea; 3 Department of Family Medicine, Seoul National University College of Medicine, 103 Daehakro, Yeongun-dong, Jongno-gu, Seoul 03080, Republic of Korea

**Keywords:** dietary fibers, psychological stress, psychological distress, depression, subjective health, mental health

## Abstract

The effect of dietary fiber intake on mental health is controversial. This study aimed to examine the association of fiber intake with mental health in Korean adults. This cross-sectional study included 11,288 participants aged ≥40 years who participated in the Korean Genome and Epidemiology Study (2004–2013). Fiber intake was assessed using a food frequency questionnaire and categorized into sex-specific quintiles. Multiple logistic regression models were used to investigate the association between the lowest quintile of fiber intake and poor mental health. Mental health was assessed using acute stress perception, the Psychosocial Well-Being Index-Short Form, self-rated health, and the Center for Epidemiological Studies–Depression Scale in Korea. Compared to those with higher fiber intake, having the lowest quintile of fiber intake was associated with higher odds of poor mental health risk, a higher risk of high-stress perception, poor psychosocial distress in males, poor psychosocial distress, and depression in females. Low fiber intake had profound negative mental health effects on males with high total energy intake and females with low total energy intake. In conclusion, there is a gender difference in the impact of total energy intake on the deleterious effect of low fiber intake on mental health.

## 1. Introduction

As the prevalence of psychological disorders has increased worldwide, maintaining a certain level of mental health has become a public health concern [1]. Mental health can be considered an interaction between psychological factors, such as acute stress and psychosocial distress, and emotional well-being factors, such as anxiety and depression. Studies have suggested that stress-related psychological factors and emotional disorders have consequential effects on adverse outcomes in several disease states, including chronic kidney disease [2], coronary heart disease [3], and cancer [4]. In addition, self-rated health has been recognized as a prognostic indicator of an individual’s quality of life, which is crucial for mental health [5]. Finding effective strategies for maintaining mental health is a public health priority.

A growing body of literature shows the significant impact of diet on mental illnesses such as depression [6]. The consumption of certain nutrients has been reported to affect psychological health. Previous observational studies in adults found that a higher intake of carbohydrates [7] and the traditional Western diet with a high amount of fat is associated with an increased risk of incident depression [8]. However, some studies have suggested that a Mediterranean diet, with sufficient vitamins and minerals, can improve mental health by reducing anxiety [9].

Dietary fiber is known for its beneficial health effects in the context of intestinal microbiome composition [10]. Previous studies have reported that the consumption of high amounts of dietary fiber is associated with a lower risk of adverse health outcomes, such as cardiovascular disorders [11], colorectal cancer [12], dementia [13], and mental disorders [14]. However, some studies have shown an insignificant effect of dietary fiber on one’s health status [15].

The literature examining the association of dietary fiber with mental health in sex-specific approaches has been lacking, even with the fact that the burden of mental disorders is estimated to be twofold higher for females than for males [16]. Therefore, the current study aimed to investigate the sex-specific association of dietary fiber with mental health, considering individuals’ total energy intake and level of physical activity simultaneously.

## 2. Materials and Methods

### 2.1. Study Participants

**Figure 1:**
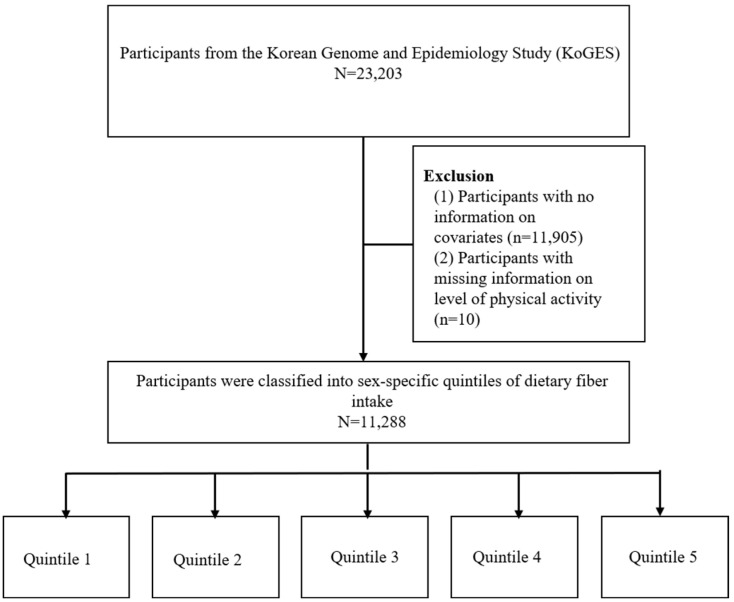
Flow diagram of study participants.

This study included participants from the Health Examinees Study, a component of the Korean Genome and Epidemiology Study (KoGES_HEXA, 2004–2013). This community-based cohort study involves participants aged 40–69 years who participated in examinations from 38 general hospitals and health examination centers. Further details of the cohort were described in a previous study [17]. Among the 23,203 baseline health checkup participants who fully completed all four types of mental health questionnaires, 11,915 with missing necessary information were excluded; ultimately, 11,288 participants were included in the study (Figure 1). The participants were categorized separately based on the quintiles of dietary fiber intake in males and females. The lowest quintile of dietary fiber intake ranged from 0.62 to 3.76 (g/day) and 0.25 to 3.59 (g/day) in men and women, respectively. All participants voluntarily enrolled in the KoGES study and provided written informed consent [18]. The current research was authorized by the Institutional Review Board of Seoul National University Hospital (IRB No. E-2406-028-1540). The ethics committee approved a waiver for the requirement of written informed consent due to HEXA data being publicly open for use and the nature of an anonymous dataset.

### 2.2. Outcome Measurement

Acute stress, defined as stress within the past month, was assessed using one item of the Korean-translated Brief Encounter Psychosocial Instrument (BEPSI-K) [19]. The questionnaire item was assessed on a three-point Likert scale ranging from ‘not at all’ to ‘frequently present’. The reliability of the BEPSI-K was demonstrated by a Cronbach alpha of 0.71.

The Psychosocial Well-Being Index short-form (PWI-SF) survey was used to assess the extent of psychosocial distress [20]. The validated PWI-SF consists of 18 measures that assess self-confidence, depression, anxiety, sleep disorders, social performance, general well-being, and vitality. The measurement instrument used for each topic is a four-point Likert scale, with a total score range of 0 to 54 points. The participants were instructed to respond to inquiries regarding alterations in items during the past few weeks and were categorized based on their overall PWI-SF score, with higher scores indicating higher levels of distress. Prior studies have shown the validity and reliability of the PWI-SF [21]. The PWI-SF has been used to assess the levels of psychosocial distress in different populations, including patients with chronic kidney disease [22], those with stroke [23], and cancer survivors [24]. In the present study, we defined poor psychosocial distress as a PWI-SF score ≥ 15 in males and ≥18 in females, the highest tertiles.

Self-rated health was assessed using the following question: “How would you rate your general health status?” [5]. The five possible answers were dichotomized as good (excellent, good, or fair) or poor (poor or very poor).

Depressive symptoms were assessed using the Korean version of the Center for Epidemiologic Studies–Depression (CES-D-K) Scale, which has demonstrated high reliability [25]. The present study defined probable depression as a score ≥ 16, validated in previous studies [26].

### 2.3. Dietary Assessment

The dietary intake of the participants was assessed using a 106-item semi-quantitative food frequency questionnaire (FFQ), which has been described in detail in previous studies [27]. The validity and reproducibility of the FFQ were evaluated, and the daily total energy and nutrient intake, including fiber, protein, fat, and carbohydrates in grams per day, were calculated from the CAN-Pro 2.0 nutrient database, developed by the Korean Nutrition Society [28]. Dietary fiber was divided into sex-specific quintiles, and a low consumption of dietary fiber was defined as the lowest quintile.

### 2.4. Baseline Measurements

Individuals were classified as having hypertension, diabetes mellitus, or dyslipidemia if they answered ‘yes’ to the question ‘Have you ever received a diagnosis from a doctor?’. Trained personnel measured anthropometric data using standardized protocols and instruments. Height (m) and weight (kg) were measured using a digital scale, and the body mass index (BMI) (kg/m^2^) was calculated. The participants provided information about smoking, drinking, exercise habits, and the level of physical activity using a health-related behavioral questionnaire. Smoking status and alcohol intake were divided into three categories: never, former, and current. A current smoker was defined as someone who had smoked at least 100 cigarettes in their lifetime and is currently smoking. Participants were asked about their alcohol intake within the last year. Individuals who consumed alcohol at least once a month were categorized as current drinkers [29]. Physical activity was divided into two categories: regular exercise (defined as engaging in physical activity for at least three times per week with at least moderate intensity) and inactivity. The level of physical activity was divided into four categories according to the classification of total weekly aerobic physical activity: inactive (no activity beyond baseline), less active (activity beyond baseline but <150 min/week), active (150–300 min/week), and highly active (>300 min/week) [30]. In the present study, those who answered highly active were considered to have a high level of physical activity, while others had a lower level of physical activity. The basal metabolic rate (BMR), which reflects the energy metabolism necessary to maintain vital functions at rest, was calculated using the Harris–Benedict equation [31]. Information on socioeconomic status was collected via a questionnaire that included monthly household income, divided into two groups: less than 3 million Korean won (KRW) and >KRW 3 million. Subjects’ blood samples were obtained via the antecubital vein following a 12 h overnight fast. The samples were then enzymatically analyzed using a Chemistry Analyzer (ADVIA 1650 and 1800, Siemens, Tarrytown, NY, USA).

### 2.5. Statistical Analyses

The participants’ demographic and clinical features were reported using descriptive statistics according to sex and quintiles of dietary fiber consumption. Continuous variables are presented as the mean and standard deviation (SD). If the distribution was not normal, the median (interquartile range) value was determined using the Kruskal–Wallis H test. Categorical variables were described as counts and percentages, and Pearson’s chi-squared and Fisher’s exact tests were used to test the differences in ratios.

Multivariate logistic regression analysis was conducted with categorized binary variables of poor mental health, including acute stress, self-rated health (SRH), PWI-SF, and CES-D, as the dependent variables and the lowest quintile of dietary fiber consumption as the independent variable, after adjusting for confounders. Model 1 was unadjusted; Model 2 was adjusted for age, BMI, smoking habits, alcohol intake, regular exercise, income, hypertension, diabetes, and dyslipidemia; and Model 3 was adjusted for the variables from Model 2 and total energy intake.

Stratified analysis according to a BMI of 25 kg/m^2^, sex-specific mean/median age, total energy intake (low and high), BMR (low and high) based on sex-specific median levels, and level of physical activity (low and high) was conducted to compare the association between dietary fiber consumption and mental health. The stratified model was then adjusted for the same covariates. Interaction models of BMI, age, total energy intake, BMR, and level of physical activity were analyzed on the association between dietary fiber intake and mental health. All analyses were performed using R software (version 4.1.2, R Foundation for Statistical Computing, Vienna, Austria), with statistical significance at *p* < 0.05.

## 3. Results

The baseline characteristics of the study participants by sex are shown in Table 1. All four mental health scores (stress, SRH, PWI-SF, and CES-D) were worse in females than in males.

The baseline characteristics of the study participants across sex-specific quintiles of dietary fiber consumption are shown in Supplementary Table S1. Participants with higher dietary fiber consumption tended to exercise regularly, have a higher income, and have a higher total energy intake. The group with higher dietary fiber consumption was more likely to have higher total energy intakes and carbohydrate intakes, whereas the carbohydrate–energy ratio, calculated as the percentage of energy intake from carbohydrates to the total energy intake, was lower. Regarding the aspect of mental health questionnaires, the participants with the lowest quintile of dietary fiber consumption have a higher prevalence of answering “frequently suffered from acute stress,” “very unhealthy self-rated health,” and higher total PWI-SF and CES-D scores.

The mental health questionnaire scores were dichotomized into high and low levels, with high levels corresponding to poor mental health. The lowest quintile of dietary fiber intake was associated with high-stress perception and poor psychosocial distress in males with odds ratios (OR) (95% confidence intervals (CI)) of 1.43 (1.20, 1.69) and 1.46 (1.21, 1.75), respectively (Table 2). In contrast, the lowest quintile of dietary fiber intake was associated with poor psychosocial distress and depression in females with ORs of 1.53 (1.35, 1.74) and 1.40 (1.14, 1.71) after adjustments.

**Figure 2:**
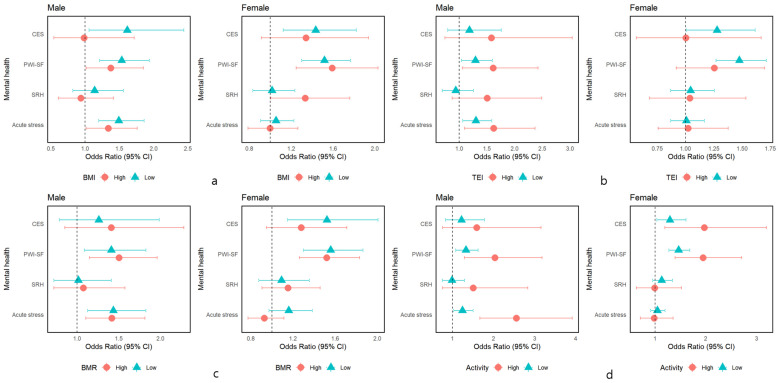
Forest plot of the logistic regression. Adjusted for age, BMI, smoking habit, alcohol intake, regular exercise, income, hypertension, diabetes, dyslipidemia, and total energy intake. Acute stress ≥ 2; SRH ≥ 4, PWI-SF ≥ 3rd tertile (≥15 in males, ≥18 in females), CES ≥ 16; BMI of 25 kg/m^2^; age of 54 and 52 years; TEI of 1807 in males, 1639 in females, BMR < 1497 in males, 1248 in females; Level of physical activity: inactive (no activity beyond baseline), less active (activity beyond baseline but fewer than 150 min a week), active (150 min to 300 min a week), and highly active (more than 300 min a week). Those who answered highly active were considered to have a high level of physical activity, and others had a lower level of physical activity in the present study. CES, Center for Epidemiologic Studies–Depression; PWI-SF, psychosocial well-being index short-form; SRH, self-rated health; Acute stress, acute stress perception; BMI, body mass index; Activity, level of physical activity; BMR, basal metabolic rate; TEI, total energy intake; 95% CI, confidence interval. (**a**) BMI, (**b**) TEI, (**c**) BMR, (**d**) Activity.

As shown in Table 3 and Figure 2, similar association patterns were observed in males after stratification by BMI, age, total energy intake, basal metabolic rate, and physical activity level. Having the lowest quintile of dietary fiber was associated with a higher risk of acute stress perception and psychosocial distress, while the odds of the lowest quintile of dietary fiber with a risk of acute stress perception and psychosocial distress were potentiated in males with high total energy intake and high level of activity from 1.30 (1.06–1.59) to 1.62 (1.10–2.37), from 1.29 (1.04–1.60) to 1.61 (1.06–2.42), from 1.24 to 2.55, and from 1.31 to 2.03, respectively. Interestingly, the impact of low fiber intake on poor psychosocial distress and depression was significant in females with low total energy intake (1.56, 1.30–1.86; 1.52, 1.15–2.01). Additionally, the association of low fiber intake with psychosocial distress and depression was potentiated in females with high levels of physical activity from 1.46 to 1.94 and from 1.29 to 1.97, respectively. Further interaction analysis revealed no interaction of total energy intake and level of physical activity with dietary fiber intake and mental health in both males and females.

## 4. Discussion

In this cross-sectional study of the general Korean population, we evaluated the association between dietary fiber intake and mental health issues such as acute stress perception, psychosocial distress, self-rated health status, and depressive symptoms. The principal finding of this study was that low dietary fiber intake was associated with a high risk of developing acute stress and poor psychosocial distress in males and poor psychosocial distress and depressive symptoms in females. The significance of this relationship was independent of age, BMI, smoking habits, alcohol intake, physical exercise, hypertension, diabetes, dyslipidemia, income, and total energy intake. The strength of the association increased in males and females with high levels of physical activity compared to their counterparts. The adverse effect of low fiber intake on mental health increases in males with high total energy intake compared with those with low total energy intake. However, the significance was maintained in females with low total energy intake and was lost in females with high total energy intake. This study is one of the first epidemiological investigations to assess the association between low dietary fiber intake and mental health, considering the potential factors related to energy intake and the level of physical activity.

The results of this study show that low dietary fiber intake is an independent risk factor for the development of high stress and depressive symptoms in Korean adults. This finding is consistent with those of several previous studies. Previous studies have reported that high fiber intake protects against depression and anxiety in adults with adequate physical activity [32]. Another longitudinal study found that high fiber intake was inversely correlated with the development of new depressive symptoms in Taiwanese people over 10 years [15]. A meta-analysis concluded that fiber intake is beneficial for reducing stress, even in healthy adults without depression [11]. A recent longitudinal study of relatively young Korean adults reported that males with a high dietary fiber intake had a relatively lower risk of developing depressive symptoms [33]. Taken together, these findings suggest that high fiber intake is beneficial for the mental health of adults, whereas low fiber intake increases the risk of developing poor mental health.

Dietary fiber intake is known to improve the gut microbiota environment, and improved intestinal flora composition plays a positive role in serotonin synthesis through the gut–brain axis [34]. Immunomodulation through fermented dietary fibers improves inflammation, which is a basic pathway in depression and stress [35]. The short-chain fatty acids, which are fermentation products of food components, play a role in shaping the gut environment and physiology of the colon, thus improving digestibility [36]. The total energy intake was linearly associated with ATP-dependent gastric emptying and digestion [37]. The beneficial role of dietary fiber against postprandial hyperglycemia might also decrease oxidative stress and improve insulin resistance, thus improving mental health [38].

Combining low fiber consumption with high levels of physical activity further increases the risk of poor mental health in males and females. This finding is also in line with the findings of a previous study showing that fruit and vegetable consumption, the primary source of dietary fiber, with high leisure-time physical activity (LTPA) further reduced the likelihood of developing subsequent depressive symptoms beyond LPTA alone, and insignificant impacts of vegetable and fruit consumption alone were reported [15]. The importance of sufficient fiber intake should be emphasized, to be accompanied by high levels of physical activity.

In the present study, there was a sex difference in the effect of total energy intake on the association between low fiber intake and poor mental health. The adverse impact of low fiber intake on mental health was significant in females with low total energy intake but not in those with high total energy intake. As previously described, appropriate fiber intake benefits mental health in males, more prominently observed in those with a BMI ≥ 25 who are reported to have high total energy intake [33]. However, the development of stress and depressive symptoms increased in females with a low total energy intake. This study’s results partially coincided with those of our previous study, which demonstrated that the energy intake–energy expenditure balance, defined as the difference between daily energy intake and energy expenditure, is negatively associated with depression in Korean females, while no significance was shown in males [39]. When the total calorie intake is low or physical activity is high, there is a greater likelihood of experiencing a negative energy imbalance. In such cases, insufficient caloric intake and low fiber intake are more likely to have detrimental effects on mental health. This result indicates that adequate dietary fiber intake may be especially important in females with a low total energy intake [40].

The possible underlying biological mechanisms can be explained by sex differences in muscle substrate metabolism [41]. Males have larger fiber cross-sectional areas that show more type II fiber characteristics, whereas females have smaller fibers that show more type I characteristics [42]. Type II muscle fibers rely more on carbohydrates than type I fibers, which rely more on fat as a fuel source. Thus, the adverse effects of insufficient fiber intake, highly complex substances with non-digestible carbohydrates, cause insufficient energy sources in males, causing a greater adverse impact in males compared to women [43]. There are also sex-related differences in gastrointestinal motility, that is, a slower gastric emptying rate in females, which is highly affected by total energy intake [44]. Slower digestion, which causes food retention, is associated with gastrointestinal tract inflammation and neuroinflammation, leading to poor mental health [45]. Sufficient energy intake, leading to ATP production, enhanced digestibility, and the availability of an ample fuel source (fat) in females, can counterbalance the negative impact of low fiber intake on digestibility in females. This is associated with the lack of a significant correlation between low fiber intake and poor mental health in females with high total energy intake. According to the Korean Ministry of Health and Welfare, males tend to have a higher energy intake than energy requirements, whereas females have a lower energy intake than energy requirements [46]. Thus, an insufficient total caloric intake with low fiber intake likely has a negative impact on the maintenance of regular physical activity and digestion, ultimately affecting mental health. Considering an individual’s energy balance simultaneously with dietary fiber intake can generally provide more information than no consideration. However, further studies are required to confirm these findings.

This study has several strengths. This study included participants from a well-established sample of middle- and older-aged Korean adults. The sex-stratified analysis avoided the potential impact of sex-specific dietary patterns on the association between variables. This study assessed mental health using four different kinds of previously validated methods. However, this study had some limitations that warrant further discussion. A history of depression or treatment with medication could not be addressed because of limited access to data. The nature of dietary fibers, such as soluble and insoluble fibers, cannot be categorized owing to limited information. The generalization should be cautious due to a single race-based study, and the genetic factors that differ between different races may affect the impact of low fiber intake on mental health [47,48]. Moreover, it was difficult to ascertain a causal relationship between dietary fiber and mental health, given the cross-sectional nature of the study. Future intervention studies are required to consolidate these findings and enhance their clinical usefulness.

## 5. Conclusions

This is the first large-scale study to reveal the relationship between dietary fiber and mental health, considering total energy intake and the level of physical activity. Lower dietary fiber intake was associated with poor mental health in both males and females. This finding underscores the importance of dietary recommendations for high fiber intake, as well as the different approaches necessary for males and females, considering the level of physical activity and total energy intake needed to maintain mental health. However, further intervention studies are required to examine the causal association between dietary fiber intake and mental health.

## Figures and Tables

**Table 1:** Characteristics of participants.

Sex	Male	Female	*p*
	(N = 4112)	(N = 7176)	
Age	54.0 [47.0; 61.0]	52.0 [47.0; 58.0]	<0.001 *
BMI			<0.001 *
Underweight	40 (1.0%)	133 (1.9%)	
Normal	1180 (28.7%)	3029 (42.2%)	
Overweight	1248 (30.4%)	1912 (26.6%)	
Obese	1644 (40.0%)	2102 (29.3%)	
Total energy (kcal/day)	1806.5 [1544.1; 2159.2]	1639.2 [1361.0; 1962.5]	<0.001 *
Protein (gram/day)	58.8 [47.3; 74.9]	52.7 [41.5; 66.7]	<0.001 *
Fat (gram/day)	27.2 [19.3; 38.5]	22.6 [15.5; 32.0]	<0.001 *
Carbohydrate (gram/day)	321.9 [281.4; 377.5]	301.3 [248.9; 351.4]	<0.001 *
Carbohydrate (%)	72.0 [67.4; 75.8]	73.2 [68.6; 77.3]	<0.001 *
Fiber (gram/day)	5.5 [4.1; 7.1]	5.3 [3.9; 7.0]	0.001 *
Smoking habit			<0.001 *
Never	1026 (25.0%)	6947 (96.8%)	
Ex-smoker	1774 (43.1%)	91 (1.3%)	
Current	1312 (31.9%)	138 (1.9%)	
Alcohol intake			<0.001 *
Never	768 (18.7%)	4753 (66.2%)	
Ex	308 (7.5%)	124 (1.7%)	
Current	3036 (73.8%)	2299 (32.0%)	
Regular exercise			<0.001 *
No	1759 (42.8%)	3396 (47.3%)	
Yes	2353 (57.2%)	3780 (52.7%)	
Hypertension	943 (22.9%)	1186 (16.5%)	<0.001 *
Diabetes	379 (9.2%)	352 (4.9%)	<0.001 *
Dyslipidemia	414 (10.1%)	614 (8.6%)	0.008 *
Income			<0.001 *
<300 KRW	2282 (55.5%)	4321 (60.2%)	
≥300 KRW	1830 (44.5%)	2855 (39.8%)	
hs-CRP (mg/dL)	0.1 [0.0; 0.1]	0.0 [0.0; 0.1]	<0.001 *
Stress			<0.001 *
No	2716 (66.1%)	3777 (52.6%)	
Intermittently	1201 (29.2%)	2781 (38.8%)	
Frequently	195 (4.7%)	618 (8.6%)	
SRH			<0.001 *
Very healthy	134 (3.3%)	124 (1.7%)	
Healthy	1824 (44.4%)	2465 (34.4%)	
Normal	1643 (40.0%)	3269 (45.6%)	
Unhealthy	487 (11.8%)	1265 (17.6%)	
Very unhealthy	24 (0.6%)	53 (0.7%)	
PWI-SF	12.0 [10.0; 18.0]	15.0 [11.0; 21.0]	<0.001 *
CES-D			<0.001 *
<16	3879 (94.3%)	6496 (90.5%)	
≥16	233 (5.7%)	680 (9.5%)	

* Significance at *p* < 0.05; BMI, body mass index; KRW, Korean won; SRH, self-rated health; PWI-SF, psychosocial well-being index short-form; CES-D, Center for Epidemiologic Studies–Depression. BMI: underweight, <18 kg/m^2^; normal, 18–23 kg/m^2^; overweight, 23–25 kg/m^2^; obese, >25 kg/m^2^. The proportions of carbohydrates were calculated by multiplying carbohydrate intake (g/day) by 4 kcal/total energy intake (kcal/day) and then multiplying by 100.

**Table 2:** Comparison of poor mental health by the lowest quintile of fiber intake (multiple logistic regression).

	Lowest Fiber Consumption					
	Model 1		Model 2		Model 3	
	OR	95% CI	OR	95% CI	OR	95% CI
Male						
Stress (≥2)	1.33 *	1.13–1.55	1.31 *	1.12–1.54	1.43 *	1.20–1.69
SRH (≥4)	1.13	0.90–1.41	1.09	0.86–1.37	1.06	0.82–1.36
PWI-SF (≥3rd tertile)	1.54 *	1.31–1.82	1.51 *	1.27–1.79	1.46 *	1.21–1.75
CES-D (≥16)	1.49 *	1.10–2.01	1.43 *	1.05–1.93	1.33	0.95–1.86
Female						
Stress (≥2)	1.09	0.97–1.22	1.04	0.92–1.17	1.04	0.92–1.18
SRH (≥4)	1.28 *	1.11–1.47	1.18 *	1.02–1.37	1.11	0.95–1.31
PWI-SF (≥3rd tertile)	1.73 *	1.54–1.95	1.64 *	1.45–1.84	1.53 *	1.35–1.74
CES-D (≥16)	1.61 *	1.35–1.93	1.44 *	1.20–1.73	1.40 *	1.14–1.71

* Significance at *p* < 0.05; Model 1: unadjusted. Model 2: adjusted for age, BMI, smoking habit, alcohol intake, regular exercise, income, hypertension, diabetes, and dyslipidemia. Model 3: adjusted for model 2 and total energy intake. SRH, self-rated health; PWI-SF, psychosocial well-being index short-form; CES-D, Center for Epidemiologic Studies–Depression.

**Table 3:** Stratified regression results according to BMI, age, energy intake, basal metabolic rate, and physical activity.

	Q1			*p* for Interaction	Q1			*p* for Interaction
		OR	95% CI			OR	95% CI	
	Male				Female			
BMI	<25				<25			
Stress		1.49 *	1.19–1.86	0.649		1.05	0.91–1.23	0.644
SRH		1.14	0.82–1.56	0.529		1.02	0.83–1.24	0.330
PWI-SF		1.53 *	1.21–1.94	0.733		1.52 *	1.30–1.77	0.826
CES-D		1.62 *	1.06–2.44	0.109		1.43 *	1.12–1.82	0.963
	≥25				≥25			
Stress		1.34 *	1.01–1.76			1.00	0.79–1.27	
SRH		0.94	0.61–1.41			1.33	1.00–1.76	
PWI-SF		1.37 *	1.02–1.85			1.59 *	1.25–2.03	
CES-D		0.98	0.54–1.72			1.34	0.92–1.94	
Age	<54				<52			
Stress		1.39 *	1.09–1.78	0.164		0.95	0.79–1.14	0.530
SRH		0.96	0.65–1.40	0.245		1.24	0.96–1.58	0.633
PWI-SF		1.39 *	1.07–1.81	0.130		1.43 *	1.19–1.73	0.152
CES-D		1.42	0.88–2.24	0.867		1.36	1.00–1.84	0.591
	≥54				≥52			
Stress		1.46 *	1.14–1.86			1.15	0.96–1.37	
SRH		1.15	0.81–1.61			1.07	0.87–1.32	
PWI-SF		1.52 *	1.17–1.98			1.66 *	1.38–1.99	
CES-D		1.21	0.74–1.95			1.47 *	1.12–1.92	
TEI	<1807				<1639			
Stress		1.30 *	1.06–1.59	0.360		1.01	0.87–1.17	0.775
SRH		0.94	0.69–1.26	0.191		1.04	0.87–1.26	0.601
PWI-SF		1.29 *	1.04–1.60	0.701		1.47 *	1.27–1.71	0.132
CES-D		1.18	0.79–1.76	0.725		1.28 *	1.01–1.62	0.114
	≥1807				≥1639			
Stress		1.62 *	1.10–2.37			1.02	0.76–1.38	
SRH		1.51	0.87–2.49			1.04	0.68–1.53	
PWI-SF		1.61 *	1.06–2.42			1.25	0.92–1.70	
CES-D		1.58	0.74–3.04			1.00	0.56–1.67	
BMR	<1497				<1248			
Stress		1.44 *	1.13–1.83	0.665		1.16	0.97–1.39	0.157
SRH		1.02	0.72–1.41	0.525		1.09	0.88–1.36	0.761
PWI-SF		1.41 *	1.09–1.83	0.470		1.56 *	1.30–1.86	0.760
CES-D		1.26	0.79–1.99	0.867		1.52 *	1.15–2.01	0.696
	≥1497				≥1248			
Stress		1.42 *	1.11–1.81			0.93	0.77–1.11	
SRH		1.07	0.72–1.57			1.15	0.91–1.46	
PWI-SF		1.50 *	1.15–1.96			1.52 *	1.26–1.83	
CES-D		1.41	0.85–2.28			1.28	0.95–1.71	
Activity	Low				Low			
Stress		1.24 *	1.03–1.50	0.035 *		1.05	0.91–1.20	0.589
SRH		0.99	0.75–1.29	0.639		1.13	0.95–1.34	0.563
PWI-SF		1.32 *	1.08–1.62	0.440		1.46 *	1.27–1.68	0.343
CES-D		1.22	0.83–1.78	0.868		1.29 *	1.03–1.61	0.279
	High				High			
Stress		2.55 *	1.67–3.91			0.98	0.71–1.36	
SRH		1.50	0.76–2.84			0.99	0.63–1.52	
PWI-SF		2.03 *	1.29–3.18			1.94 *	1.40–2.71	
CES-D		1.59	0.76–3.15			1.97 *	1.19–3.19	

* Significance at *p* < 0.05; Adjusted for age, BMI, smoking habits, alcohol intake, physical exercise, hypertension, diabetes, dyslipidemia, income, and total energy intake. Q, quintile; BMI, body mass index; SRH, self-rated health; PWI-SF, psychosocial well-being index short-form; CES-D, Center for Epidemiologic Studies–Depression.

## Data Availability

The data presented in this study are available on request from the corresponding author due to legal restriction on sharing information. Researchers are required to submit ethics approval, and a detailed research plan to the Korea Disease Control and Prevention Agency (KDCA) to request access to the data.

## Supplementary Materials

The following supporting information can be downloaded at: https://www.mdpi.com/article/10.3390/nu16162583/s1, Table S1: Characteristics of participants according to quintiles of dietary fiber intake.
