# Supplementary material for: Gender Difference in the Impact of Total Energy Intake on the Association between Low Fiber Intake and Mental Health in Middle-Aged and Older Adults
Source: Nutrients. 2024 Aug 6;16(16):2583. doi: 10.3390/nu16162583 (PMC11357506; doi:10.3390/nu16162583)
Supplement: Supplementary file 1 [file nutrients-16-02583-s001.zip › nutrients-3114901-supplementary.pdf]

Supplementary Table S1. Characteristics of participants according to quintiles of dietary fiber intake.

Fiber intake	Q1	Q2	Q3	Q4	Q5	p
	(0.62/3.76)	(3.77/4.94)	(4.95/6.03)	(6.04/7.57)	(7.58/37.77)	
	(N=823)	(N=823)	(N=822)	(N=822)	(N=822)	
Male						
Age	54.0 [47.0; 61.0]	55.0 [48.0; 62.0]	53.0 [47.0; 61.0]	54.0 [46.0; 60.0]	53.0 [45.0; 61.0]	<0.001*
BMI	24.3 [22.6; 26.0]	24.2 [22.6; 25.9]	24.3 [22.6; 25.9]	24.4 [22.7; 26.3]	24.5 [22.9; 26.2]	0.08
Total energy intake (kcal/day)	1488.0 [1297.2; 1684.0]	1659.7 [1471.8; 1867.0]	1823.2 [1612.5; 2088.6]	1977.6 [1725.1; 2286.4]	2314.7 [1967.3; 2710.4]	<0.001*
Protein (gram/day)	42.7 [36.1; 51.7]	52.0 [44.3; 60.4]	59.9 [50.4; 70.3]	67.6 [57.2; 79.4]	84.0 [67.9; 101.4]	<0.001*
Fat (gran/day)	19.5 [13.7; 26.6]	23.6 [17.4; 31.3]	27.7 [20.4; 37.2]	32.2 [23.8; 43.0]	39.2 [28.2; 53.8]	<0.001*
Carbohydrate (gram/day)	277.9 [239.4; 307.9]	302.4 [269.3; 332.9]	325.1 [293.8; 365.8]	344.2 [306.4; 395.4]	393.0 [342.0; 466.3]	<0.001*
Carbohydrate (%)	74.5 [70.3; 77.9]	73.2 [69.3; 76.4]	71.9 [67.8; 75.6]	70.7 [65.9; 74.7]	69.5 [64.6; 74.0]	<0.001*
Fiber (gram/day)	3.0 [2.5; 3.4]	4.4 [4.1; 4.6]	5.5 [5.2; 5.7]	6.7 [6.3; 7.1]	9.2 [8.2; 10.7]	<0.001*
Smoking habit						0.472
Never	222 (27.0%)	192 (23.3%)	213 (25.9%)	198 (24.1%)	201 (24.5%)	
Ex-smoker	327 (39.7%)	366 (44.5%)	365 (44.4%)	358 (43.6%)	358 (43.6%)	
Current	274 (33.3%)	265 (32.2%)	244 (29.7%)	266 (32.4%)	263 (32.0%)	
Alcohol intake						0.09
Never	174 (21.1%)	148 (18.0%)	171 (20.8%)	144 (17.5%)	131 (15.9%)	
Ex	63 (7.7%)	56 (6.8%)	55 (6.7%)	61 (7.4%)	73 (8.9%)	
Current	586 (71.2%)	619 (75.2%)	596 (72.5%)	617 (75.1%)	618 (75.2%)	
Regular exercise						0.013*
No	392 (47.6%)	353 (42.9%)	353 (42.9%)	325 (39.5%)	336 (40.9%)	
Yes	431 (52.4%)	470 (57.1%)	469 (57.1%)	497 (60.5%)	486 (59.1%)	
Hypertension	181 (22.0%)	183 (22.2%)	198 (24.1%)	195 (23.7%)	186 (22.6%)	0.81
Diabetes	74 (9.0%)	82 (10.0%)	83 (10.1%)	78 (9.5%)	62 (7.5%)	0.381
Dyslipidemia	84 (10.2%)	81 (9.8%)	87 (10.6%)	87 (10.6%)	75 (9.1%)	0.852
Income						0.226
<300KRW	464 (56.4%)	469 (57.0%)	473 (57.5%)	437 (53.2%)	439 (53.4%)	
≥300KRW	359 (43.6%)	354 (43.0%)	349 (42.5%)	385 (46.8%)	383 (46.6%)	
hs-CRP (mg/dL)	0.1 [0.0; 0.1]	0.1 [0.0; 0.1]	0.1 [0.0; 0.1]	0.1 [0.0; 0.1]	0.1 [0.0; 0.1]	0.019*
Stress						0.011*
No	501 (60.9%)	543 (66.0%)	547 (66.5%)	562 (68.4%)	563 (68.5%)	
Intermittently	271 (32.9%)	245 (29.8%)	240 (29.2%)	231 (28.1%)	214 (26.0%)	
Frequently	51 (6.2%)	35 (4.3%)	35 (4.3%)	29 (3.5%)	45 (5.5%)	
SRH						0.105
Very healthy	21 (2.6%)	27 (3.3%)	22 (2.7%)	30 (3.6%)	34 (4.1%)	
Healthy	342 (41.6%)	342 (41.6%)	366 (44.5%)	405 (49.3%)	369 (44.9%)	

Normal	349 (42.4%)	354 (43.0%)	329 (40.0%)	295 (35.9%)	316 (38.4%)	
Unhealthy	103 (12.5%)	97 (11.8%)	100 (12.2%)	88 (10.7%)	99 (12.0%)	
Very unhealthy	8 (1.0%)	3 (0.4%)	5 (0.6%)	4 (0.5%)	4 (0.5%)	
PWI-SF	14.0 [11.0; 20.0]	13.0 [10.0; 18.0]	12.0 [10.0; 17.0]	12.0 [9.0; 16.0]	12.0 [9.0; 17.0]	<0.001*
CES-D						0.04*
<16	761 (92.5%)	777 (94.4%)	773 (94.0%)	789 (96.0%)	779 (94.8%)	
≥16	62 (7.5%)	46 (5.6%)	49 (6.0%)	33 (4.0%)	43 (5.2%)	

Fiber intake	Q1	Q2	Q3	Q4	Q5	p
	(0.25/3.59)	(3.60/4.74)	(4.75/5.87)	(5.88/7.55)	(7.56/34.69)	
	(N=1436)	(N=1435)	(N=1435)	(N=1435)	(N=1435)	
Female						
Age	52.0 [46.5; 59.0]	52.0 [46.5; 58.0]	52.0 [47.0; 58.0]	52.0 [47.0; 58.0]	52.0 [47.0; 57.0]	0.549
BMI	23.2 [21.5; 25.2]	23.4 [21.7; 25.3]	23.4 [21.7; 25.4]	23.5 [21.7; 25.4]	23.4 [21.8; 25.4]	0.309
Total energy intake (kcal/day)	1281.8 [1026.8; 1498.2]	1499.2 [1287.6; 1707.6]	1645.5 [1426.6; 1878.2]	1815.8 [1577.8; 2057.4]	2079.9 [1795.2; 2466.6]	<0.001*
Protein (gram/day)	37.1 [30.1; 44.1]	46.5 [39.3; 54.1]	53.1 [44.7; 62.5]	60.1 [51.0; 71.3]	74.9 [61.1; 91.5]	<0.001*
Fat (gram/day)	14.8 [10.1; 20.9]	19.8 [14.1; 26.9]	22.9 [16.6; 30.9]	26.0 [19.4; 34.2]	32.5 [23.3; 46.6]	<0.001*
Carbohydrate (gram/day)	241.2 [185.7; 285.3]	282.4 [229.5; 315.0]	302.3 [258.3; 339.0]	330.6 [284.7; 368.1]	369.2 [319.6; 428.2]	<0.001*
Carbohydrate (%)	75.5 [70.4;79.2]	74.0 [69.5;77.5]	73.0 [68.7;77.0]	72.5 [68.4;76.4]	71.1 [66.1;75.5]	<0.001*
Fiber (gram/day)	2.9 [2.4; 3.3]	4.2 [3.9; 4.5]	5.3 [5.0; 5.6]	6.6 [6.2; 7.0]	9.1 [8.2; 10.8]	<0.001*
Smoking habit						0.005*
Never	1370 (95.4%)	1385 (96.5%)	1386 (96.6%)	1408 (98.1%)	1398 (97.4%)	
Ex-smoker	29 (2.0%)	16 (1.1%)	21 (1.5%)	10 (0.7%)	15 (1.0%)	
Current	37 (2.6%)	34 (2.4%)	28 (2.0%)	17 (1.2%)	22 (1.5%)	
Alcohol intake						0.395
Never	933 (65.0%)	956 (66.6%)	946 (65.9%)	953 (66.4%)	965 (67.2%)	
Ex	33 (2.3%)	21 (1.5%)	22 (1.5%)	18 (1.3%)	30 (2.1%)	
Current	470 (32.7%)	458 (31.9%)	467 (32.5%)	464 (32.3%)	440 (30.7%)	
Regular exercise						<0.001*
No	746 (51.9%)	750 (52.3%)	662 (46.1%)	641 (44.7%)	597 (41.6%)	
Yes	690 (48.1%)	685 (47.7%)	773 (53.9%)	794 (55.3%)	838 (58.4%)	
Hypertension	251 (17.5%)	245 (17.1%)	238 (16.6%)	235 (16.4%)	217 (15.1%)	0.503
Diabetes	95 (6.6%)	63 (4.4%)	65 (4.5%)	60 (4.2%)	69 (4.8%)	0.018*
Dyslipidemia	129 (9.0%)	117 (8.2%)	110 (7.7%)	121 (8.4%)	137 (9.5%)	0.417
Income						<0.001*
<300 KRW	919 (64.0%)	877 (61.1%)	857 (59.7%)	875 (61.0%)	793 (55.3%)	

≥300 KRW	517 (36.0%)	558 (38.9%)	578 (40.3%)	560 (39.0%)	642 (44.7%)	
hs-CRP (mg/dL)	0.1 [0.0; 0.1]	0.0 [0.0; 0.1]	0.1 [0.0; 0.1]	0.1 [0.0; 0.1]	0.0 [0.0; 0.1]	0.306
Stress						0.065
No	732 (51.0%)	769 (53.6%)	717 (50.0%)	795 (55.4%)	764 (53.2%)	
Intermittently	566 (39.4%)	541 (37.7%)	588 (41.0%)	540 (37.6%)	546 (38.0%)	
Frequently	138 (9.6%)	125 (8.7%)	130 (9.1%)	100 (7.0%)	125 (8.7%)	
SRH						<0.001*
Very healthy	20 (1.4%)	24 (1.7%)	25 (1.7%)	22 (1.5%)	33 (2.3%)	
Healthy	419 (29.2%)	475 (33.1%)	488 (34.0%)	543 (37.8%)	540 (37.6%)	
Normal	689 (48.0%)	672 (46.8%)	660 (46.0%)	627 (43.7%)	621 (43.3%)	
Unhealthy	293 (20.4%)	252 (17.6%)	255 (17.8%)	231 (16.1%)	234 (16.3%)	
Very unhealthy	15 (1.0%)	12 (0.8%)	7 (0.5%)	12 (0.8%)	7 (0.5%)	
PWI-SF	17.0 [12.0; 23.0]	15.0 [11.0; 21.0]	14.0 [11.0; 20.0]	13.0 [11.0; 19.0]	14.0 [10.5; 19.0]	<0.001*
CES-D						<0.001*
<16	1248 (86.9%)	1298 (90.5%)	1297 (90.4%)	1345 (93.7%)	1308 (91.1%)	
≥16	188 (13.1%)	137 (9.5%)	138 (9.6%)	90 (6.3%)	127 (8.9%)	

* Significance at $p < 0.05$; BMI, Body Mass Index; CES-D, Center for Epidemiologic Studies Depression Scale; hs-CRP, High-Sensitivity C-Reactive Protein; KRW, Korean Won; PWI-SF, Personal Wellbeing Index - Short Form; SRH, Self-Reported Health. The proportions of carbohydrates were calculated by multiplying carbohydrate intake (g/day) by 4 kcal/total energy intake (kcal/day) and then multiplying 100.
